# Does Additional Dietary Supplementation Improve Physiotherapeutic Treatment Outcome in Tendinopathy? A Systematic Review and Meta-Analysis

**DOI:** 10.3390/jcm11061666

**Published:** 2022-03-17

**Authors:** Fanji Qiu, Jinfeng Li, Kirsten Legerlotz

**Affiliations:** 1 Institute of Sport Sciences, Humboldt-Universität zu Berlin, Unter den Linden 6, 10099 Berlin, Germany; fanji.qiu@student.hu-berlin.de; 2 Department of Kinesiology, Iowa State University, Ames, IA 50011, USA; jfli@iastate.edu

**Keywords:** nutrition, tendinopathy, tendon pain, exercise therapy, meta-analysis, VISA, VAS

## Abstract

A systematic review and meta-analysis of randomized controlled trials was performed to evaluate the effects of dietary supplements in addition to physiotherapeutic treatment on pain and functional outcomes. PubMed, The Cochrane Library, Web of Science, and Embase were searched from inception to November 2021 (Prospero registration: CRD42021291951). Studies were eligible if the interventions consisted of physiotherapeutic approaches that were combined with dietary supplementation and if they reported measures of pain and/or function. Six studies were included in the meta-analysis. Standardized mean differences (SMD) and 95% confidence intervals (CI) were calculated and analysed using a Review Manager software. Subgroup analysis was performed to explore possible associations between the study characteristics and the effectiveness of the intervention. Additional dietary supplementation during physiotherapeutic treatment significantly improved the reduction in pain score (SMD = −0.74, 95% CI, −1.37 to −0.10; *p* < 0.05), while it had no effect on functional outcomes (SMD = 0.29, 95% CI, 0.00 to 0.58; *p* > 0.05). This systematic review and meta-analysis suggests that additional nutritional interventions may improve physiotherapeutic treatment outcomes in the management of tendinopathies.

## 1. Introduction

Tendinopathy is a common tendon disorder accounting for around 30% of complaints related to musculoskeletal pain and functional limitations [1]. The aetiology of this tendon pathology is multifactorial, including mechanical overuse [1,2] and metabolic disorders [3,4,5]. Usually, tendinopathies are characterized by tendon pain and impaired function, which also leads to a reduction in exercise participation in tendinopathic patients [6]. In the last few years, the incidence of tendinopathies in athletes and in the general population has increased [7], with the increases in physical activity, changes in lifestyle and side effects of medication being discussed as potentially influencing factors [7,8,9,10].

In the management of tendinopathies, a variety of physiotherapy treatment methods have been applied, including extracorporeal shock wave [11] and manual therapy [12] as well as exercise interventions such as heavy slow resistance training [13,14] and eccentric training [15,16].

In recent years, dietary supplements have been introduced as an additional therapeutic approach in the treatment of tendinopathies, and their positive curative effects have been reported for both the general population and athletes [17,18,19,20]. The applied dietary supplements contained a variety of micronutrients (e.g., bromelain and vitamin C) [21,22,23], which are suggested to reduce the level of inflammation [24,25,26] and to promote the healing of the tendon [21,22,27,28,29,30]. Because of the suggested anti-inflammatory effects and claimed improvements in tendon metabolism, treatments that combine dietary supplements with physiotherapy have been explored in some studies [23,31,32,33,34,35,36].

The combination treatment of dietary supplementation and physiotherapy has been evaluated in a series of clinical trials due to these compelling rationales. Considering that individual trials are unlikely to provide sufficient evidence to guide clinical practice, we attempted to objectively evaluate the potential impact of this treatment strategy in tendinopathy management, to provide a higher level of clinical evidence. Thus, we performed a systematic review and meta-analysis of randomized controlled trials to assess the effect of additional dietary supplementation in combination with physiotherapy treatment on the key outcomes of tendinopathy treatment such as pain and function in comparison to physiotherapeutic treatment alone or physiotherapeutic treatment with placebo.

## 2. Methods

This systematic review and meta-analysis adhered to the PRISMA statement [37] and was registered at Prospero (CRD42021291951) in the international prospective register of systematic reviews in November 2021.

### 2.1. Eligibility Criteria

#### 2.1.1. Participants

Studies were eligible if participants were aged under 70 years. No restrictions on gender, ethnicity, or sport participation were applied.

#### 2.1.2. Interventions

Studies were eligible if the interventions consisted of any kind of physiotherapeutic approaches (e.g., eccentric training, shockwave or laser therapy), which were combined with dietary supplementation. The intervention programs were required to last at least 4 weeks and were conducted in any setting (e.g., laboratory, home, gym).

#### 2.1.3. Comparisons

The control groups for comparison were subjected to physiotherapy alone or to physiotherapy and placebo treatment.

#### 2.1.4. Outcomes

Studies were eligible if they reported at least one outcome of interest, which were measures of pain and/or function. We selected NRS (Numerical Rating Scales) and VAS (Visual Analogue Scales) scores as assessment of pain. These two measures were synthesized, as recent studies have shown that NRS and VAS scores, both ranging from 0 to 10, correspond to each other [38]. In addition, we assessed reliable and valid functional outcome scores [39,40,41,42,43,44], including the Victorian Institute of Sports Assessment for Achilles or Patellar Tendon (VISA-A and VISA-P), the Shoulder Pain and Disability Index (SPADI) and the Ankle–Hindfoot Scale (AHS).

#### 2.1.5. Study Design

Inclusion criteria: studies were randomized controlled trials (RCTs) published in English. Exclusion criteria: (1) duplicate publications; (2) literature review papers; (3letters to the editor; (4) abstracts published in conference proceedings; (5) animal model studies. Articles with the full text unavailable were also excluded.

### 2.2. Search Strategy and Study Selection

We selected relevant studies published before November 2021, by searching PubMed, Cochrane, Embase, and Web of Science databases. We applied the English language, and the search keywords included population (e.g., tendinopathy) and intervention (e.g., dietary supplement). For PubMed, we used the Mesh Database, combined Mesh terms and entry terms, and made some adjustments in other databases. Moreover, we applied the filters: randomized controlled trial (publication types), randomized (title/abstract), or placebo (title/abstract) when appropriate. The precise search strategy is described in the supplemental file (Table S1). All the results were collated by a reference management tool (Endnote X9, Thomson Reuters, NY, USA), and duplicates were removed. We considered all potentially eligible studies. In addition, we performed a manual search using the references of key articles. Two authors (F.Q., J.L.) independently reviewed study titles and abstracts retrieved, to include the articles that satisfied the requirements, and then read the full texts for final eligibility. Any disagreements were resolved by consensus with the third reviewer (K.L.).

### 2.3. Data Extraction

Data extraction and results compilation were performed by two independent reviewers (F.Q., J.L.), and data were extracted into Microsoft Excel. In case of disagreement, a third researcher (K.L.) intervened. As no original data on of pain scores and functional outcomes were provided in the studies of Juhasz et al. [31] and Praet et al. [35], the values were extracted from the reported graphs using Graph digitizer software (Digitizelt, Braunschweig, Germany). Data extracted from the studies included the following information: lead author, year of publication; demographic characteristics (age, gender); sample size in the experimental and control groups; study groups; intervention characteristics: type of tendinopathy (e.g., Achilles tendon, rotator cuff), intervention duration (in weeks); type of population (athletic or non-athletic), type of physiotherapy (e.g., strength training, shock wave or laser therapy) and reported outcomes (pain and function); symptom duration; time points of measurement; pharmaceutical company supplying the nutritional supplements; composition of supplements; assumed effect of supplement.

### 2.4. Quality Assessment and Risk of Bias

The included studies were assessed according to the Cochrane Risk of Bias tool [45]. The following domains were assessed: (1) random sequence generation; (2) concealment of allocation; (3) blinding of participants, investigators, and assessors; (4) blinding of outcome assessment; (5) incomplete outcome data processed; (6) selective reporting bias; (7) other bias. The domains were given a rating of low (+), unclear (?), or high risk (−) of bias.

No funnel plots were graphed for any of the comparisons, given that the number of studies in each comparison was less than 10 [46]. For sensitivity analysis [47], we evaluated the robustness of the pooled results by exclusion-by-exclusion, removing one study data from the pooled analysis at a time to evaluate the consistency between the pooled results of the remaining studies and the pooled results of all studies.

### 2.5. Statistical Analysis

The meta-analysis was performed using the Review Manager software version 5.4 (The Cochrane Collaboration, Copenhagen, Denmark). As the Victorian Institute of Sports Assessment–Achilles questionnaire (VISA-A), the Shoulder Pain and Disability Index (SPADI) and the Ankle–Hindfoot Scale (AHS) were not assessed by using consistent forms of measurement, standardized mean differences (SMD) were applied. All results are expressed as mean difference (MD) with standard deviation (SD) and were reported with 95% confidence intervals (CIs). The interquartile ranges and 95% CIs with standard errors were calculated with the appropriate formulas to convert them to means and standard errors (SDs) [48]. Random-effects and fixed-effects models were used to estimate pooled effects by accounting for study differences and weighting each study accordingly. We used the Cochran Q test [49] and I^2^ testing [50] to assess the magnitude of the heterogeneity between studies, with I^2^ greater than 50% or a *p* value at or less than 0.10 in Q test, indicating the presence of moderate-to-high heterogeneity. Subgroup analyses were conducted to explore possible associations between the study characteristics and the effectiveness of the intervention. Study characteristics included type of tendinopathy (Achilles tendon, other type), intervention duration (≤8 weeks or >8 weeks), type of physiotherapy (exercise therapy, ultrasound (US), extracorporeal shockwave therapy (ESWT)) and type of population (athletic, non-athletic). For subgroup analysis, statistical significance was established at an alpha level of 0.05.

## 3. Results

### 3.1. Search Yield

**Figure 1:**
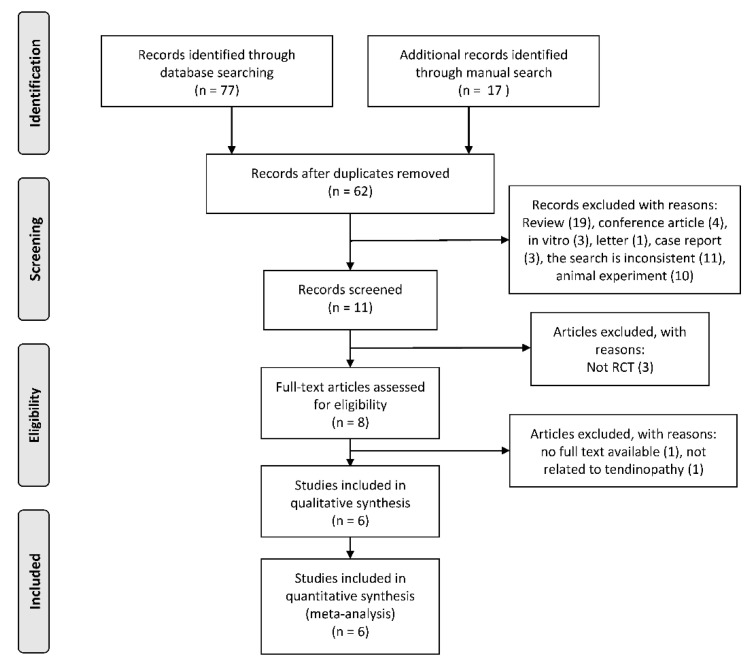
Study selection process (according to the PRISMA guidelines).

We identified a total of 94 potentially eligible articles during the trial selection process. Sixty-two articles remained for screening after removing duplicates. After screening titles and abstracts, six articles [23,31,32,33,34,35,36] were included in the meta-analysis (Figure 1). From all included studies, an experimental group of 123 people and a control group of 118 people were evaluated in this meta-analysis.

### 3.2. Characteristics of Included Studies

Summed up, the six studies (Table 1) included 241 participants, with the mean (SD) age ranging from 14.8 (1.6) to 55.8 (13.2) years. The six studies applied six different commercially available dietary supplements (Table 2). The duration of the intervention in the six articles ranged from 4 to 12 weeks, while the symptom duration ranged from 4 weeks to 3 years. The time from the start of the intervention to the end point of the intervention or follow-up respectively ranged from 32 days to 1 year. Five of the included studies examined indices of pain [23,31,32,34,36], and four examined indices of function [23,34,35,36]. The interventions in all six studies compared dietary supplementation combined with physiotherapy and placebo combined with physiotherapy or physiotherapy alone. Two trials combined dietary supplementation with ESWT (extracorporeal shockwave therapy) or therapeutic ultrasound; four trials combined dietary supplementation with exercise therapy. In the study by Balius et al. [23], before being randomly assigned to the intervention group, the subjects were classified according to the pathology model of Cook and Purdam [51] as suffering from reactive or degenerative tendinopathy. Data of six experimental groups were included in the pooled analysis of the pain score, and data of five groups were included in the pooled analysis of the functional outcome. 

### 3.3. Assessment of Risk of Bias

According to the Cochrane Risk of Bias tool, one article was evaluated as high risk of bias in randomization for grouping patients according to age (Table 3). Group allocation was clearly stated in half of the included articles [32,35,36]. Three studies were evaluated as low risk in terms of blinding. Regarding detection bias, three articles were evaluated as low risk. One study was assessed as high risk in reporting bias, as the overall effect of treatment was not reported. Two articles were evaluated as high risk of bias due to the financial support from the pharmaceutical company that was supplying the nutritional supplement.

Further sensitivity analysis and exclusion-by-exclusion of the literature led to significant changes in the results of the pain score and functional outcomes, which were caused by the studies by Notarnicola et al. [34] and Sandford et al. [36], respectively. Although high heterogeneity (I2 = 77%) was found in the pain score, all five trials showed a significant reduction in the pain score with additional dietary supplementation.

### 3.4. Meta-Analysis

#### 3.4.1. Analysis of Pain Score at Rest

**Figure 2:**
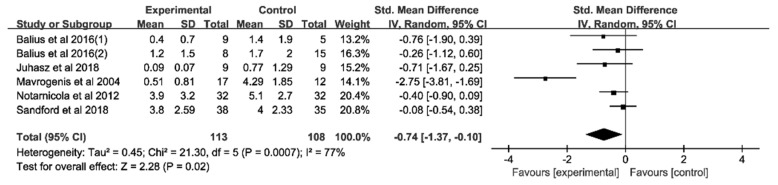
Forest plot of the meta-analysis on pain score at rest. Pain score measurement include NRS and VAS scales.

Five experiments with a total of 221 participants provided data on pain score at rest. The pooled analysis of five trials (Figure 2) revealed that the combination of dietary supplementation and physiotherapy led to a greater mean reduction in pain score than physiotherapy alone (SMD = −0.74, 95%CI, −1.37 to −0.10). In the subgroup analysis (Table 4) of types of tendinopathy, intervention duration, physiotherapy type, and type of population, we observed no significant effects. 

#### 3.4.2. Analysis of Functional Outcomes

**Figure 3:**
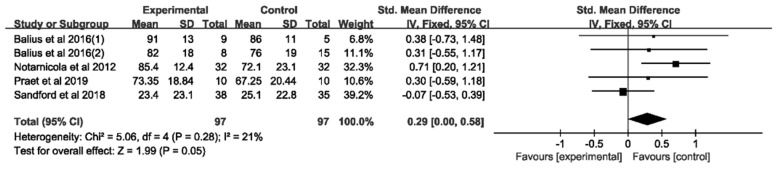
Forest plot of the meta-analysis on functional outcomes. Functional outcomes include VISA-A, SPADI and AHS.

Four RCTs with a total of 194 participants provided data on functional outcomes. The pooled analysis (Figure 3) revealed that the combination of dietary supplementation and physiotherapy led to no superior improvement in functional outcomes compared to physiotherapy alone (SMD = 0.29, 95%CI, 0.00 to 0.58). In the subgroup analysis (Table 4), we observed a greater effect of physiotherapy alone in Achilles tendinopathy (*p* = 0.005).

## 4. Discussion

In the management of tendinopathy, the reduction of pain and the improvement of function are the aims of different treatment methods [6,52,53,54,55,56,57,58]. Within this context, a novel strategy that applied dietary supplementation additional to physiotherapy was evaluated. Our meta-analysis has shown that additional nutritional interventions can reduce pain further than physiotherapy alone, while a significant improvement in function could not be detected.

Additional nutritional interventions may be an effective strategy to improve pain reduction in patients with tendinopathies. The mechanism by which the ingestion of nutritional supplements improves tendinopathy symptoms may be linked to the capacity of certain ingredients in affecting inflammation. While all six RCTs included in our meta-analysis applied a different supplement, five studies [23,31,32,34,36] applied supplements which contained substances of known anti-inflammatory function, and all six papers explained assumed effects with a reduction in inflammation or inflammatory markers.

Inflammation is strongly associated with pain production [59,60]. In addition, it may play an important role in the development of tendinopathies [14,61]. Therefore, downregulation of inflammation levels induced by anti-inflammatory ingredients could be a potential mechanism to consider in tendinopathy management. The pain-reducing effect of nutritional interventions addressing inflammation has been shown for a variety of supplements in a variety of tendon related pathologies. In patients with lateral epicondylitis and shoulder and Achilles tendinopathy [62], Tendisulfur^®^ supplementation for 60 days led to a drop in VAS, possibly due to the downregulation of NF-κB, TNF-α and IL-6. Furthermore, in patients subjected to supraspinatus tendon repair [33], two months of supplementation with Tendisulfur^®^ resulted in a significant reduction in the VAS score, which was assumed to be related to the inhibition the NF-κB pathway by boswellia serrata and curcuma longa within the supplement. In patients with shoulder, knee, elbow and hip tendinopathy, a recent study [63] has shown that one-month supplementation with curcuminoids and boswellia serrata extracts significantly reduced the VAS score. They explained this effect by the anti-inflammatory activity of curcuma longa and boswellia serrata extracts. In patients subjected to rotator cuff tear repair [64], a three-month supplementation with tenosan significantly reduced the pain scores in patients. The authors suggested that the supplement may have resulted in an inhibitory effect on the generation of bradykinin and catabolic signalling pathways in tenocytes [64]. In patients with Achilles tendinopathy, a 12-week intervention with a supplement containing collagen peptide type-1, low molecular weight chondroitin sulphate, sodium hyaluronate, and vitamin C led to a significant decrease in the VAS score [65]. The authors suggested that the therapeutic effects may be associated with an improvement in the glycosaminoglycan composition and collagen synthesis of the tendon by the ingredients of the supplementation [65].

The pain-reducing effects of nutritional supplements have also been observed in patients with other musculoskeletal injuries. In patients with mild knee pain, using low-fat yoghurt supplemented with rooster comb extract for 12 weeks led to a significant improvement in pain, possibly caused by a decrease in inflammation [66]. In patients with fibromyalgia syndrome (FMS), 12 weeks supplementation with an extract of salmon’s milt reduced the pain severity score significantly [67]. This may have been caused by an inhibition of inflammation, as indicated by dropped serum levels of the tumour necrosis factor (TNF) and substance P [67]. While further research is needed to explore the effectiveness of the specific components within the supplementation, it appears that nutritional interventions, which contain substances addressing inflammation, have the potential to speed up the recovery of musculoskeletal injuries and to improve the therapeutic response in terms of pain reduction [23,31,32,33,34,35,36,62,68]. 

Despite a reduction in pain, nutritional supplementation during exercise therapy does not seem to further enhance functional outcomes in tendinopathy patients. While two comparative studies [28,62] have reported significant improvements in functional scales and pain in patients with Achilles, patellar and rotator cuff tendinopathy after receiving nutritional supplementation, the results of the RCTs do not point in the same direction. Our subgroup analysis of RCTs investigating Achilles tendinopathy was in favour of physiotherapy alone. A reduction in pain does not necessarily translate into an improvement in function, which has been reported in various studies. In patients with Achilles tendinopathy, treatment with nonsteroidal anti-inflammatory medication (NSAIDs) for 1 week resulted in an improvement in VAS, while no significant changes in the VISA-A were detected [69]. In patients with temporomandibular joint osteoarthritis [70], the ingestion of a supplement containing glucosamine, chondroitin sulphate, and methylsulfonylmethane for 3 months led to a reduction in VAS but not to a significant change in mandibular mobility, suggesting that the supplementation was not able to trigger an adequate adaptive response to improve joint function. In adolescent swimmers with muscle damage induced by high-intensity interval swimming, supplementation with whey protein caused a significant decrease in pain score, while no beneficial effect on swimming performance was observed [71]. While a reduction in pain may be a necessary prerequisite to improve function, these studies suggest that pain relief is by no means automatically associated with an improvement in function or performance. 

Although human and in vitro studies have shown that vitamin C has the potential of stimulating collagen synthesis [18] and Tendoactive^®^ has the potential to inhibit the NF-κB-mediated IL-1ß catabolic signalling pathway in tendon cells [24], it appears that the acceleration of collagen synthesis is not necessarily reflected in scales measuring function.

In addition, it needs to be considered, that by reducing inflammation, the adaptive response could be impaired. Anti-inflammatory supplements have been found to suppress the production of prostaglandin E2 (PGE2) [72,73], and such changes can affect matrix remodelling and thus lead to impairments in tendon healing [74,75]. In healthy adults, one week of NSAID administration has been shown to cause a reduction in PGE2 and to minimize the adaptive increase in collagen synthesis in human patellar tendons induced by exercise [76]. Furthermore, not all patients suffering from tendinopathy present with signs of inflammation such as increased IL6 levels [14], and suppression of inflammation in those patients may not be warranted. This may explain why nutritional supplements aiming to reduce inflammation work poorly in patients with degenerative tendinopathy [23]. It may also explain why NSAID treatment, as a commonly applied approach to reduce musculoskeletal pain, does not necessarily lead to symptom improvement in tendinopathic patients. In a randomized controlled trial with 70 Achilles tendinopathy patients, a 28-day long treatment with the NSAID piroxicam did neither improve pain nor function compared to the placebo-treated group [77]. In this context, it needs to be highlighted that long-term oral NSAID intake may result in significant adverse effects, e.g., related to the gastrointestinal system [78]. The advantage of nutritional supplementation as an alternative to NSAID treatment may therefore be inherent in the lower risk to induce significant adverse side effects. However, when using additional nutritional supplementation as an intervention, it is noteworthy to pay attention to how functional parameters are affected by pain and how the reduction of inflammation may affect tendon metabolism.

## 5. Limitations

Overall, the number of studies included in this meta-analysis is small. As a result, subgroup analyses of some study characteristics were prevented, and no funnel plot could be graphed because the number of studies was small such that the test power was too low to distinguish chance from real asymmetry [46]. Second, included studies embraced a diverse range of dietary supplements with different ingredients and physiotherapy types and with a wide range of intervention durations (6 weeks to 6 months), which makes it difficult to draw any definitive conclusions on the use of combination treatment in tendinopathies. Third, potential factors such as funding from pharmaceutical companies and lack of random sequence generation might have affected the risk of bias.

The study by Balius et al. [23] was assessed to be at “high risk” of bias in five of seven evaluated areas, which may affect the final results. Since two studies [23,34] reported receiving funding from pharmaceutical companies, it cannot be excluded that the results of current research may be influenced by financial bias. In sensitivity analysis, the poor robustness of the pooled results in pain scores resulted from the methodology in the Notarnicola et al. [34] study, with only two of seven evaluated areas assessed as “low risk”, while the poor robustness of the pooled results in functional outcomes was caused by the sample size in the Sandford et al. [36] study, which was the highest within all included studies. The high heterogeneity of the Mavrogenis et al. [32] study stems from the diverse types of tendinopathies they explored within one study. Compared to exploring only one type of tendinopathy, including diverse types of tendinopathies, will affect the accuracy of the meta-analysis results.

## 6. Conclusions

In the management of tendinopathy, dietary supplementation in addition to physiotherapeutic treatment may be an effective strategy to reduce pain. However, more high-quality methodology RCTs with a sufficiently large sample size are required to confirm and establish a more definitive conclusion. With regard to the efficacy of dietary supplements combined with physiotherapy, studies have frequently used supplements which contain diverse components and which are supposed to fulfil multiple functions such as an improvement of collagen fibre organization [79] and downregulation of the level of oxidative stress and inflammation [24,27], limiting the evaluation of each single ingredient’s effect. Future studies should aim to limit the kinds of ingredients in dietary supplements for more targeted results.

Clinical implications of additional nutritional interventions in the management of tendinopathies are the potential to allow for a reduction of the use of painkillers and faster recovery.

## Figures and Tables

**Table 1:** Characteristics of included studies.

First Author (Year)	Tendon Investigated	Study Groups	Sample Size (n)	Type of Population	Mean Age (Years; Mean ± SD)	Intervention Duration (Weeks)	Symptom Duration (Months)	Time Points of Measurement
Balius et al. (2016)	Achilles tendon (mid-portion)	MCVC + EC	17	non-athletic	43.5 ± 14.5	12	>3	baseline, 6 and 12 w
		EC	20		38.9 ± 6.6			
Juhasz et al. (2018)	Musculus flexor hallucis longus	Creatine	9	athletic	15.5 ± 1.4	6	1–1.5	2, 4 and 6 w
		Placebo	9		14.8 ± 1.6			
Mavrogenis et al. (2004)	Patellar & several upper body tendons *	EFA, AO and US	17	athletic	31	5	>3	8, 16, 24 and 32 d
		Placebo and US	14		32			
Notarnicola et al. (2012)	Achilles tendon (insertional)	ESWT and tenosan	32	non-athletic	55.8 ± 13.2	8	>6	2 and 6 m
		ESWT and placebo	32					
Praet et al. (2019)	Achilles tendon (mid-portion)	TENDOFORTE + EccEx	10	non-athletic	45.3 ± 6.4	12	18	3 and 6 m
		Placebo + EccEx	10		42.0 ± 9.4			
Sandford et al. (2018)	Rotator cuff	PUFAs	38	non-athletic	52.2 ± 12.0	8	>3	8 w, 3, 6 and 12 m
		Placebo	35		52.0 ± 16.2			

d, days; m, months; w, weeks; AO, antioxidants; EC/EccEx, eccentric exercise; EFA, essential fatty acids; ESWT, extracorporeal shockwave therapy; MCVC, mucopolisaccharides, type I collagen, and vitamin C; PUFAs, polyunsaturated fatty acids; US, ultrasound; *, upper body tendons: supraspinatus, biceps, lateral epicondyle extensormedial epicondyle flexor, and infraspinatus.

**Table 2:** Details of dietary Supplements.

First Author (Year)	Dietary Supplements (Company)	Ingredients of Dietary Supplement	Assumed Effect of Supplement
Balius et al. (2016)	**TendoActive** (Bioiberica SA, Palafolls, Spain)	mucopolysaccharides, collagen type I, vitamin C	suppression of NF-κB mediated IL-1ß catabolic signalling pathways in tenocytes
Juhasz et al. (2018)	**Micronized Cr monohydrate** (BioTech, Inc., Ft. Lauderdale, FL, USA)	Cr monohydrate, dextrose, and vitamin C	reduction of inflammatory markers
Mavrogenis et al. (2004)	**Bio-Sport** (Pharma Nord ApS, Vejle, Denmark)	EPA, DHA and GLA. selenium, zinc, vitamin A, vitamin B6, vitamin C and vitamin E	reduction of inflammation caused by essential fatty acids and antioxidants
Notarnicola et al. (2012)	**Tenosan** (Agave s.r.l., Prato, Italy)	arginine-L-alpha-ketoglutarate, MSM, hydrolysed collagen type I, Vinitrox, bromelain, and vitamin C	stimulation of metabolism and proliferation; reduction of inflammation and neoangiogenesis
Praet et al. (2019)	**TENDOFORTE^®^** (GELITA AG, Eberbach, Germany)	Hydrolysed specific collagen peptides	stimulation of collagen type I and III, proteoglycans and elastin content synthesis by sCPs; reduction of TNF-alpha, matrix metalloproteases and stimulation of tissue inhibitors of metalloproteinases by Glycine
Sandford et al. (2018)	**MaxEPA** (Seven Seas Ltd., Hull, UK)	EPA, DHA and vitamin E acetate	reduction of inflammation

Cr, creatine; DHA, docosahexaenoic acid; EPA, eicosapentaenoic acid; GLA, gamma-linolenic acid; sCP, specific collagen peptides.

**Table 3:** Risk of bias assessment for RCTs according to the Cochrane Risk of Bias Tool.

First Author (year)	Random Sequence Generation (Selection Bias)	Allocation Concealment (Selection Bias)	Blinding of Participants and Personnel (Performance Bias)	Blinding of Outcome Assessment (Detection Bias)	Incomplete Outcome Data (Attrition Bias)	Selective Reporting (Reporting Bias)	Other Bias
Balius et al. (2016)	+	-	-	-	?	-	-
Juhasz et al. (2018)	?	?	?	?	+	+	+
Mavrogenis et al. (2004)	+	+	+	+	-	+	?
Notarnicola et al. (2012)	-	?	+	?	-	+	-
Praet et al. (2019)	+	+	+	+	+	+	+
Sandford et al. (2018)	+	+	+	+	+	+	+

-, high risk of bias; +, low risk of bias; ?, unclear risk of bias, as paper contained insufficient information to permit judgement.

**Table 4:** Results of subgroup analysis.

Study Characteristics	Studies	Pain at Rest	*p* Value	Functional Outcomes		
		Effect Size (95%CI)		Studies	Effect Size (95%CI)	*p* Value
Type of Tendinopathy						
Achilles Tendon	2	−0.41 (−0.83, 0.00)	>0.05	3	0.53 (0.16, 0.90)	0.005
Other Type	2	−1.72 (−3.72, 0.28)	>0.05	-	-	-
Intervention Duration						
≤8 weeks	4	−0.88 (−1.78, 0.08)	>0.05	2	0.28 (−0.06, 0.62)	>0.05
>8 weeks				2	0.32 (−0.22, 0.86)	>0.05
Type of physiotherapy						
Exercise therapy	3	−0.26 (−0.62, 0.09)	>0.05	3	0.09 (−0.26, 0.44)	> 0.05
ESWT/US	2	−1.53 (−3.83, 0.78)	>0.05	-	-	-
Type of population						
Athletic	2	−1.72 (−3.72, 0.28)	>0.05	-	-	-
Non-athletic	3	−0.26 (−0.57, 0.05)	>0.05	4	0.29 (0.00, 0.58)	>0.05

## Data Availability

Not applicable.

## Supplementary Materials

The following supporting information can be downloaded at: https://www.mdpi.com/article/10.3390/jcm11061666/s1, Table S1: search strategy.

## References

[1] Fusini F., Bisicchia S., Bottegoni C., Gigante A., Zanchini F., Busilacchi A. (2016). Nutraceutical supplement in the management of tendinopathies: A systematic review. Muscles Ligaments Tendons J..

[2] Xu Y., Murrell G.A.C. (2008). The basic science of tendinopathy. Clin. Orthop. Relat. Res..

[3] Skovgaard D., Siersma V.D., Klausen S.B., Visnes H., Haukenes I., Bang C.W., Bager P., Gravare Silbernagel K., Gaida J., Magnusson S.P. (2021). Chronic hyperglycemia, hypercholesterolemia, and metabolic syndrome are associated with risk of tendon injury. Scand. J. Med. Sci. Sports.

[4] Squier K., Scott A., Hunt M.A., Brunham L.R., Wilson D.R., Screen H., Waugh C.M. (2021). The effects of cholesterol accumulation on Achilles tendon biomechanics: A cross-sectional study. PLoS ONE.

[5] Ranger T.A., Wong A.M.Y., Cook J.L., Gaida J.E. (2015). Is there an association between tendinopathy and diabetes mellitus? A systematic review with meta-analysis. Br. J. Sports Med..

[6] Millar N.L., Silbernagel K.G., Thorborg K., Kirwan P.D., Galatz L.M., Abrams G.D., Murrell G.A.C., McInnes I.B., Rodeo S.A. (2021). Tendinopathy. Nat. Rev. Dis. Prim..

[7] Maffulli N., Wong J., Almekinders L.C. (2003). Types and epidemiology of tendinopathy. Clin. Sports Med..

[8] Knapik J.J., Pope R. (2020). Achilles Tendinopathy: Pathophysiology, Epidemiology, Diagnosis, Treatment, Prevention, and Screening. J. Spec. Oper. Med..

[9] Sobhani S., Dekker R., Postema K., Dijkstra P.U. (2012). Epidemiology of ankle and foot overuse injuries in sports: A systematic review. Scand. J. Med. Sci. Sports.

[10] Bidell M.R., Lodise T.P. (2016). Fluoroquinolone-Associated Tendinopathy: Does Levofloxacin Pose the Greatest Risk?. Pharmacotherapy.

[11] Mani-Babu S., Morrissey D., Waugh C., Screen H., Barton C. (2014). The Effectiveness of Extracorporeal Shock Wave Therapy in Lower Limb Tendinopathy: A Systematic Review. Am. J. Sports Med..

[12] Desjardins-Charbonneau A., Roy J.-S., Dionne C.E., Fremont P., MacDermid J.C., Desmeules F. (2015). The Efficacy of Manual Therapy for Rotator Cuff Tendinopathy: A Systematic Review and Meta-analysis. J. Orthop. Sports Phys. Ther..

[13] Beyer R., Kongsgaard M., Hougs Kjær B., Øhlenschlæger T., Kjær M., Magnusson S.P. (2015). Heavy Slow Resistance Versus Eccentric Training as Treatment for Achilles Tendinopathy: A Randomized Controlled Trial. Am. J. Sports Med..

[14] Radovanović G., Wolfarth B., Legerlotz K. (2019). Interleukin-6 levels drop after a 12 week long physiotherapeutic intervention in patients with Achilles tendinopathy—A pilot study. Transl. Sports Med..

[15] Woodley B.L., Newsham-West R.J., Baxter G.D. (2007). Chronic tendinopathy: Effectiveness of eccentric exercise. Br. J. Sports Med..

[16] van der Vlist A.C., Winters M., Weir A., Ardern C.L., Welton N.J., Caldwell D.M., Verhaar J.A.N., de Vos R.J. (2021). Which treatment is most effective for patients with Achilles tendinopathy? A living systematic review with network meta-analysis of 29 randomised controlled trials. Br. J. Sports Med..

[17] Maughan R.J., Burke L.M., Dvorak J., Larson-Meyer D.E., Peeling P., Phillips S.M., Rawson E.S., Walsh N.P., Garthe I., Geyer H. (2018). IOC consensus statement: Dietary supplements and the high-performance athlete. Br. J. Sports Med..

[18] Shaw G., Lee-Barthel A., Ross M.L., Wang B., Baar K. (2017). Vitamin C–enriched gelatin supplementation before intermittent activity augments collagen synthesis. Am. J. Clin. Nutr..

[19] Clark K.L., Sebastianelli W., Flechsenhar K.R., Aukermann D.F., Meza F., Millard R.L., Deitch J.R., Sherbondy P.S., Albert A. (2008). 24-Week study on the use of collagen hydrolysate as a dietary supplement in athletes with activity-related joint pain. Curr. Med. Res. Opin..

[20] Zdzieblik D., Oesser S., Gollhofer A., König D. (2017). Improvement of activity-related knee joint discomfort following supplementation of specific collagen peptides. Appl. Physiol. Nutr. Metab..

[21] Aiyegbusi A.I., Duru F.I., Awelimobor D., Noronha C.C., Okanlawon A.O. (2010). The role of aqueous extract of pineapple fruit parts on the healing of acute crush tendon injury. Niger. Q. J. Hosp. Med..

[22] Kao W.W.-Y., Flaks J.G., Prockop D.J. (1976). Primary and secondary effects of ascorbate on procollagen synthesis and protein synthesis by primary cultures of tendon fibroblasts. Arch. Biochem. Biophys..

[23] Balius R., Álvarez G., Baró F., Jiménez F., Pedret C., Costa E., Martínez-Puig D. (2016). A 3-Arm Randomized Trial for Achilles Tendinopathy: Eccentric Training, Eccentric Training Plus a Dietary Supplement Containing Mucopolysaccharides, or Passive Stretching Plus a Dietary Supplement Containing Mucopolysaccharides. Curr. Ther. Res. Clin. Exp..

[24] Shakibaei M., Buhrmann C., Mobasheri A. (2011). Anti-inflammatory and anti-catabolic effects of TENDOACTIVE® on human tenocytes in vitro. Histol. Histopathol..

[25] Bassit R.A., Curi R., Costa Rosa L.F. (2008). Creatine supplementation reduces plasma levels of pro-inflammatory cytokines and PGE2 after a half-ironman competition. Amino Acids.

[26] DePhillipo N.N., Aman Z.S., Kennedy M.I., Begley J.P., Moatshe G., Laprade R.F. (2018). Efficacy of Vitamin C Supplementation on Collagen Synthesis and Oxidative Stress After Musculoskeletal Injuries: A Systematic Review. Orthop. J. Sports Med..

[27] Ömeroğlu S., Peker T., Türközkan N., Ömeroğlu H. (2009). High-dose vitamin C supplementation accelerates the Achilles tendon healing in healthy rats. Arch. Orthop. Trauma Surg..

[28] Puig D.M., Arquer A., García M., Laucirica J.A., Rius M., Blàvia M., Fontserè J., Hernández C., Boluda J., Kranjcec T. (2014). The efficacy and safety of oral mucopolysaccharide, type I collagen and vitamin C treatment in tendinopathy patients. Apunt. Med. De L’esport.

[29] Chisari E., Rehak L., Khan W.S., Maffulli N. (2019). Tendon healing in presence of chronic low-level inflammation: A systematic review. Br. Med. Bull..

[30] Fu S.C., Cheng W.-H., Cheuk Y.-C., Mok T.-Y., Rolf C., Yung S.-H., Chan K.-M. (2013). Development of vitamin C irrigation saline to promote graft healing in anterior cruciate ligament reconstruction. J. Orthop. Transl..

[31] Juhasz I., Kopkane J.P., Hajdu P., Szalay G., Kopper B., Tihanyi J. (2018). Creatine Supplementation Supports the Rehabilitation of Adolescent Fin Swimmers in Tendon Overuse Injury Cases. J. Sports Sci. Med..

[32] Mavrogenis S., Johannessen E., Jensen P., Sindberg C. (2004). The effect of essential fatty acids and antioxidants combined with physiotherapy treatment in recreational athletes with chronic tendon disorders: A randomised, double-blind, placebo-controlled study. Phys. Ther. Sport.

[33] Merolla G., Dellabiancia F., Ingardia A., Paladini P., Porcellini G. (2015). Co-analgesic therapy for arthroscopic supraspinatus tendon repair pain using a dietary supplement containing Boswellia serrata and Curcuma longa: A prospective randomized placebo-controlled study. Musculoskelet. Surg..

[34] Notarnicola A., Pesce V., Vicenti G., Tafuri S., Forcignanò M., Moretti B. (2012). SWAAT Study: Extracorporeal Shock Wave Therapy and Arginine Supplementation and Other Nutraceuticals for Insertional Achilles Tendinopathy. Adv. Ther..

[35] Praet S.F.E., Purdam C.R., Welvaert M., Vlahovich N., Lovell G., Burke L.M., Gaida J.E., Manzanero S., Hughes D., Waddington G. (2019). Oral Supplementation of Specific Collagen Peptides Combined with Calf-Strengthening Exercises Enhances Function and Reduces Pain in Achilles Tendinopathy Patients. Nutrients.

[36] Sandford F.M., Sanders T., Wilson H., Lewis J.S. (2018). A randomised controlled trial of long-chain omega-3 polyunsaturated fatty acids in the management of rotator cuff related shoulder pain. BMJ Open Sport Exerc. Med..

[37] Page M.J., McKenzie J.E., Bossuyt P.M., Boutron I., Hoffmann T.C., Mulrow C.D., Shamseer L., Tetzlaff J.M., Akl E.A., Brennan S.E. (2021). The PRISMA 2020 statement: An updated guideline for reporting systematic reviews. Int. J. Surg..

[38] Hjermstad M.J., Fayers P.M., Haugen D.F., Caraceni A., Hanks G.W., Loge J.H., Fainsinger R., Aass N., Kaasa S., European Palliative Care Research Collaborative (EPCRC) (2011). Studies Comparing Numerical Rating Scales, Verbal Rating Scales, and Visual Analogue Scales for Assessment of Pain Intensity in Adults: A Systematic Literature Review. J. Pain Symptom Manag..

[39] Dawson J., Hill G., Fitzpatrick R., Carr A. (2001). The benefits of using patient-based methods of assessment. Medium-term results of an observational study of shoulder surgery. J. Bone Jt. Surg. Br. Vol..

[40] Angst F., Schwyzer H.-K., Aeschlimann A., Simmen B.R., Goldhahn J. (2011). Measures of adult shoulder function: Disabilities of the Arm, Shoulder, and Hand Questionnaire (DASH) and its short ver-sion (QuickDASH), Shoulder Pain and Disability Index (SPADI), American Shoulder and Elbow Surgeons (ASES) Society standardized shoulder assessment form, Constant (Murley) Score (CS), Simple Shoulder Test (SST), Oxford Shoulder Score (OSS), Shoulder Disability Questionnaire (SDQ), and Western Ontario Shoulder Instability Index (WOSI). Arthritis Care Res..

[41] Bot S.D., Terwee C.B., van der Windt D.A., Bouter L.M., Dekker J., de Vet H.C. (2004). Clinimetric evaluation of shoulder disability questionnaires: A systematic review of the literature. Ann. Rheum. Dis..

[42] Breckenridge J.D., McAuley J.H. (2011). Shoulder Pain and Disability Index (SPADI). J. Physiother..

[43] Murphy M., Rio E., Debenham J., Docking S., Travers M., Gibson W. (2018). Evaluating the Progress of Mid-Portion Achilles Tendinopathy during Rehabilitation: A Review of Outcome Measures for Self- Reported Pain and Function. Int. J. Sports Phys. Ther..

[44] Kostuj T., Stief F., Hartmann K.A., Schaper K., Arabmotlagh M., Baums M.H., Meurer A., Krummenauer F., Lieske S. (2018). Using the Oxford Foot Model to determine the association between objective measures of foot function and results of the AOFAS Ankle-Hindfoot Scale and the Foot Function Index: A prospective gait analysis study in Germany. BMJ Open.

[45] Higgins J.P., Altman D.G., Gøtzsche P.C., Jüni P., Moher D., Oxman A.D., Savovic J., Schulz K.F., Weeks L., Sterne J.A. (2011). The Cochrane Collaboration’s tool for assessing risk of bias in randomised trials. BMJ.

[46] Sterne J.A., Sutton A.J., Ioannidis J.P., Terrin N., Jones D.R., Lau J., Carpenter J., Rücker G., Harbord R.M., Schmid C.H. (2011). Recommendations for examining and interpreting funnel plot asymmetry in meta-analyses of randomised controlled trials. BMJ.

[47] Higgins J.P.T., Thomas J., Chandler J., Cumpston M., Li T., Page M.J., Welch V.A. (2019). Cochrane Handbook for Systematic Reviews of Interventions.

[48] Borenstein M. (2009). Introduction to Meta-Analysis.

[49] Bowden J., Tierney J.F., Copas A.J., Burdett S. (2011). Quantifying, displaying and accounting for heterogeneity in the meta-analysis of RCTs using standard and generalised Q statistics. BMC Med. Res. Methodol..

[50] Higgins J.P., Thompson S.G., Deeks J.J., Altman D.G. (2003). Measuring inconsistency in meta-analyses. BMJ.

[51] Alfredson H., Cook J. (2007). A treatment algorithm for managing Achilles tendinopathy: New treatment options. Br. J. Sports Med..

[52] Cardoso T.B., Pizzari T., Kinsella R., Hope D., Cook J.L. (2019). Current trends in tendinopathy management. Best. Pract. Res. Clin. Rheumatol..

[53] Murphy M., Travers M., Gibson W., Chivers P., Debenham J., Docking S., Rio E. (2018). Rate of Improvement of Pain and Function in Mid-Portion Achilles Tendinopathy with Loading Protocols: A Systematic Review and Longitudinal Meta-Analysis. Sports Med..

[54] Vander Doelen T., Jelley W. (2020). Non-surgical treatment of patellar tendinopathy: A systematic review of randomized controlled trials. J. Sci. Med. Sport.

[55] Liu X., Machado G.C., Eyles J.P., Ravi V., Hunter D.J. (2018). Dietary supplements for treating osteoarthritis: A systematic review and meta-analysis. Br. J. Sports Med..

[56] Puigdellivol J., Comellas Berenger C., Perez Fernandez M.A., Cowalinsky Millan J.M., Carreras Vidal C., Gil Gil I., Martinez Pagan J., Ruiz Nieto B., Jimenez Gomez F., Comas Figuerola F.X. (2019). Effectiveness of a Dietary Supplement Containing Hydrolyzed Collagen, Chondroitin Sulfate, and Glucosamine in Pain Reduction and Functional Capacity in Osteoarthritis Patients. J. Diet. Suppl..

[57] Dragan S., Serban M.C., Damian G., Buleu F., Valcovici M., Christodorescu R. (2020). Dietary Patterns and Interventions to Alleviate Chronic Pain. Nutrients.

[58] Agergaard A.-S., Svensson R.B., Malmgaard-Clausen N.M., Couppé C., Hjortshoej M.H., Doessing S., Kjaer M., Magnusson S.P. (2021). Clinical Outcomes, Structure, and Function Improve with Both Heavy and Moderate Loads in the Treatment of Patellar Tendinopathy: A Randomized Clinical Trial. Am. J. Sports Med..

[59] Ji R.R., Chamessian A., Zhang Y.Q. (2016). Pain regulation by non-neuronal cells and inflammation. Science.

[60] Ronchetti S., Migliorati G., Delfino D.V. (2017). Association of inflammatory mediators with pain perception. Biomed. Pharm..

[61] Legerlotz K., Jones E.R., Screen H.R., Riley G.P. (2012). Increased expression of IL-6 family members in tendon pathology. Rheumatology.

[62] Vitali M., Naim Rodriguez N., Pironti P., Drossinos A., Di Carlo G., Chawla A., Gianfranco F. (2019). ESWT and nutraceutical supplementation (Tendisulfur Forte) vs ESWT-only in the treatment of lateral epicondylitis, Achilles tendinopathy, and rotator cuff tendinopathy: A comparative study. J. Drug Assess..

[63] Henrotin Y., Dierckxsens Y., Delisse G., Seidel L., Albert A. (2021). Curcuminoids and Boswellia serrata extracts combination decreases tendinopathy symptoms: Findings from an open-label post-observational study. Curr. Med. Res. Opin..

[64] Gumina S., Passaretti D., Gurzi M.D., Candela V. (2012). Arginine L-alpha-ketoglutarate, methylsulfonylmethane, hydrolyzed type I collagen and bromelain in rotator cuff tear repair: A prospective randomized study. Curr. Med. Res. Opin..

[65] Choudhary A., Sahu S., Vasudeva A., Sheikh N.A., Venkataraman S., Handa G., Wadhwa S., Singh U., Gamanagati S., Yadav S.L. (2021). Comparing Effectiveness of Combination of Collagen Peptide Type-1, Low Molecular Weight Chondroitin Sulphate, Sodium Hyaluronate, and Vitamin-C Versus Oral Diclofenac Sodium in Achilles Tendinopathy: A Prospective Randomized Control Trial. Cureus.

[66] Morina D., Fernandez-Castillejo S., Valls R.M., Pedret A., Taltavull N., Romeu M., Giralt M., Montero M., Bernal G., Faba J. (2018). Effectiveness of a low-fat yoghurt supplemented with rooster comb extract on muscle strength in adults with mild knee pain and mechanisms of action on muscle regeneration. Food Funct..

[67] Tsilioni I., Pipis H., Freitag M.S.C., Izquierdo M.D.C., Freitag K., Theoharides T.C. (2019). Effects of an Extract of Salmon Milt on Symptoms and Serum TNF and Substance P in Patients with Fibromyalgia Syndrome. Clin. Ther..

[68] Baar K. (2019). Stress Relaxation and Targeted Nutrition to Treat Patellar Tendinopathy. Int. J. Sport Nutr. Exerc. Metab..

[69] Heinemeier K.M., Øhlenschlæger T.F., Mikkelsen U.R., Sønder F., Schjerling P., Svensson R.B., Kjaer M. (2017). Effects of anti-inflammatory (NSAID) treatment on human tendinopathic tissue. J. Appl. Physiol..

[70] Cömert Kılıç S. (2021). Does glucosamine, chondroitin sulfate, and methylsulfonylmethane supplementation improve the outcome of temporomandibular joint osteoarthritis management with arthrocentesis plus intraarticular hyaluronic acid injection. A randomized clinical trial. J. Cranio-Maxillofac. Surg..

[71] McKinlay B.J., Theocharidis A., Adebero T., Kurgan N., Fajardo V.A., Roy B.D., Josse A.R., Logan-Sprenger H.M., Falk B., Klentrou P. (2020). Effects of Post-Exercise Whey Protein Consumption on Recovery Indices in Adolescent Swimmers. Int. J. Environ. Res. Public Health.

[72] Shahnazi M., Mohammadi M., Mohaddes G., Latifi Z., Ghasemnejad T., Nouri M., Fattahi A. (2018). Dietary omega-3 and -6 fatty acids affect the expression of prostaglandin E2 synthesis enzymes and receptors in mice uteri during the window of pre-implantation. Biochem. Biophys. Res. Commun..

[73] Baugé C., Leclercq S., Conrozier T., Boumediene K. (2015). TOL19-001 reduces inflammation and MMP expression in monolayer cultures of tendon cells. BMC Complement. Altern. Med..

[74] Chan K.-M., Fu S.-C. (2009). Anti-inflammatory management for tendon injuries-friends or foes?. BMC Sports Sci. Med. Rehabil..

[75] Sauerschnig M., Stolberg-Stolberg J., Schmidt C., Wienerroither V., Plecko M., Schlichting K., Perka C., Dynybil C. (2018). Effect of COX-2 inhibition on tendon-to-bone healing and PGE2 concentration after anterior cruciate ligament reconstruction. Eur. J. Med. Res..

[76] Christensen B., Dandanell S., Kjaer M., Langberg H. (2010). Effect of anti-inflammatory medication on the running-induced rise in patella tendon collagen synthesis in humans. J. Appl. Physiol..

[77] Åström M., Westlin N. (1992). No effect of piroxicam on Achilles tendinopathy: A randomized study of 70 patients. Acta Orthop. Scand..

[78] Pattanittum P., Turner T., Green S., Buchbinder R. (2013). Non-steroidal anti-inflammatory drugs (NSAIDs) for treating lateral elbow pain in adults. Cochrane Database Syst. Rev..

[79] Jiang D., Gao P., Lin H., Geng H. (2016). Curcumin improves tendon healing in rats: A histological, biochemical, and functional evaluation. Connect. Tissue Res..

