# Supplementary material for: Does Additional Dietary Supplementation Improve Physiotherapeutic Treatment Outcome in Tendinopathy? A Systematic Review and Meta-Analysis
Source: J Clin Med. 2022 Mar 17;11(6):1666. doi: 10.3390/jcm11061666 (PMC8950117; doi:10.3390/jcm11061666)
Supplement: Supplementary file 1 [file jcm-11-01666-s001.zip › jcm-1621102-supplementary.pdf]

Supplemental File

Table S1: search strategy

Search strategy in Pubmed	Search strategy in Web of Science
((((((((((((Tendinopathy[MeSH Terms]) OR (Tendinopathies[Title/Abstract])) OR (Tendonopathy[Title/Abstract])) OR (Tendonopathies[Title/Abstract])) OR (Tendinosis[Title/Abstract])) OR (Tendinoses[Title/Abstract])) OR (Tendonosis[Title/Abstract])) OR (Tendonoses[Title/Abstract])) OR (Tendinitis[Title/Abstract])) OR (Tendinitides[Title/Abstract])) OR (Tendonitis[Title/Abstract])) OR (Tendonitides[Title/Abstract])) AND ((((((((((((((((((((Dietary Supplements[MeSH Terms]) OR (Dietary Supplement[Title/Abstract])) OR (Supplements, Dietary[Title/Abstract])) OR (Dietary Supplementations[Title/Abstract])) OR (Supplementations, Dietary[Title/Abstract])) OR (Food Supplementations[Title/Abstract])) OR (Food Supplements[Title/Abstract])) OR (Food Supplement[Title/Abstract])) OR (Supplement, Food[Title/Abstract])) OR (Supplements, Food[Title/Abstract])) OR (Nutraceuticals[Title/Abstract])) OR (Nutraceutical[Title/Abstract])) OR (Nutriceuticals[Title/Abstract])) OR (Nutriceutical[Title/Abstract])) OR (Neutraceuticals[Title/Abstract])) OR (Neutraceutical[Title/Abstract])) OR (Herbal Supplements[Title/Abstract])) OR (Herbal Supplement[Title/Abstract])) OR (Supplement, Herbal[Title/Abstract])) OR (Supplements, Herbal[Title/Abstract]))	((TS=(Tendinopathy OR Tendinopathies OR Tendonopathy OR Tendonopathies OR Tendinosis OR Tendinoses OR Tendonosis OR Tendonoses OR Tendinitis OR Tendinitides OR Tendonitis OR Tendonitides)) AND (TS=(Dietary Supplements OR Dietary Supplement OR Supplements, Dietary OR Dietary Supplementations OR Supplementations, Dietary OR Food Supplementations OR Food Supplements OR Food Supplement OR Supplement, Food OR Supplements, Food OR Nutraceuticals OR Nutraceutical OR Nutriceuticals OR Nutriceutical OR Neutraceuticals OR Neutraceutical OR Herbal Supplements OR Herbal Supplement OR Supplement, Herbal OR Supplements, Herbal))) and (LANGUAGE: (English))

Search strategy in Embase	Search strategy in Cochrane
('tendinitis'/exp OR 'hypertrophic infiltrative tendinitis' OR 'nodular tendinitis' OR 'tendinitis' OR 'tendinopathy' OR 'tendinosis' OR 'tendonitis' OR 'tendonopathy' OR 'tenonitis' OR 'tenontitis' OR 'tenositis') AND ('dietary supplement'/exp OR 'diet additive' OR 'diet supplement' OR 'dietary supplement' OR 'dietary supplements' OR 'food supplement' OR 'supplementary diet')	(MeSH descriptor: [Tendinopathy] explode all trees) AND (MeSH descriptor: [Dietary Supplements] explode all trees)
